# Temperature Decreases Spread Parameters of the New Covid-19 Case Dynamics

**DOI:** 10.3390/biology9050094

**Published:** 2020-05-03

**Authors:** Jacques Demongeot, Yannis Flet-Berliac, Hervé Seligmann

**Affiliations:** 1 Laboratory AGEIS EA 7407, Team Tools for e-Gnosis Medical & Labcom CNRS/UGA/OrangeLabs Telecom4Health, Faculty of Medicine, Université Grenoble Alpes, F-38700 La Tronche, France; varanuseremius@gmail.com; 2 Laboratory CRIStAL, UMR 9189, CNRS, Université de Lille, Campus Scientifique, Avenue Henri Poincaré, F-59655 Villeneuve d’Ascq, France; yannis.flet-berliac@univ-lille.fr; 3 The National Natural History Collections, The Hebrew University of Jerusalem, Jerusalem 91404, Israel

**Keywords:** covid-19, temperature sensitivity, weather-dependent virulence, heat inhibition

## Abstract

(1) Background: The virulence of coronavirus diseases due to viruses like SARS-CoV or MERS-CoV decreases in humid and hot weather. The putative temperature dependence of infectivity by the new coronavirus SARS-CoV-2 or covid-19 has a high predictive medical interest. (2) Methods: External temperature and new covid-19 cases in 21 countries and in the French administrative regions were collected from public data. Associations between epidemiological parameters of the new case dynamics and temperature were examined using an ARIMA model. (3) Results: We show that, in the first stages of the epidemic, the velocity of contagion decreases with country- or region-wise temperature. (4) Conclusions: Results indicate that high temperatures diminish initial contagion rates, but seasonal temperature effects at later stages of the epidemy remain questionable. Confinement policies and other eviction rules should account for climatological heterogeneities, in order to adapt the public health decisions to possible geographic or seasonal gradients.

## 1. Introduction

Two coronavirus pandemics occurred in the last 20 years, transmitted from animals to humans: (i) in 2002, the SARS-CoV virus provoked a severe acute respiratory syndrome (SARS) and spread from China, with more than 8000 cases and 774 deaths in 30 countries (about 10% mortality) [1] and (ii) in 2012, the MERS-CoV virus caused also a respiratory syndrome and spread from Saudi Arabia, with 1589 cases and 567 deaths in 26 countries (about 30% mortality) [1]. In 2019, the SARS-CoV-2 virus (called also covid-19) epidemic started in China in December in Wuhan (Hubei province) [2]. The SARS-CoV-2 and SARS-CoV genomes are very similar [3] and the spread of SARS-CoV is temperature-dependent [4]. Hence, SARS-CoV-2 is suspected, like other coronaviruses, to have a weather-dependent virulence. The changes in weather alone would not necessarily decrease numbers of confirmed cases during the whole epidemic, but warm and humid weather could make SARS-CoV-2 less transmissible and less stable, provoking a break in the chain of transmission, and hence diminishing the contagious force of the disease [5]. Ancient [1,4,5] studies, as well as characteristics of the covid-19 disease compared to past corona infections like the SARS one [3,6,7,8], suggest that the spread of covid-19 could diminish in warm weather, particularly at the start of the epidemic, and may have a low temperature threshold under which it could spread fastest. These seasonal changes could occur in exactly the same way as for other pathogens, like the common cold or influenza [9,10,11,12]. This phenomenon can be modelled and the deterministic as well as stochastic models [2,13,14,15,16,17,18,19,20,21,22,23,24,25] include potentially temperature-dependent parameters, like the contagion coefficient increasing with cold, dry weather because of faster evaporation of aerosol droplets. The present paper aims to identify such parameters from the covid-19 spread dynamics.

Section 2 describes how we collected information about the covid-19 spread since the beginning of March 2020. Then, we proposed two types of modelling in which parameters can depend on the temperature and we give a statistical method for studying the anti-correlation between the spread velocity and the external temperature. In Section 3, we give the results of the correlation analysis for all French administrative regions and for 21 countries suffering the covid-19 epidemic.

## 2. Materials and Methods

### 2.1. Epidemic Data Extraction

We used web sites giving world weather data (like https://www.weather-atlas.com or http://data.un.org/Data.aspx?d=CLINO&f=ElementCode%3A11). Analyses at country level used mean annual temperatures for that country (from https://en.wikipedia.org/wiki/List_of_countries_by_average_yearly_temperature), and mean daily temperatures for the first half of March in the administrative capital of each French administrative region.

Covid-19 spread data exist in databases such as www.who.int/emergencies/diseases/novel-coronavirus-2019, www.cdc.gov/coronavirus/2019-ncov, www.cia.gov/library/publicapublications/the-world-factbook/fields, www.worldometers.info/coronavirus, www.fr.statista.com/statistiques/1101324/morts-coronavirus-monde, www.santepubliquefrance.fr/recherche/#search=COVID-19%20:%20point%20epidemiologique&sort=date). These repositories are updated daily and offer free access to information concerning new cases, deaths and recovered cases, which is sufficient to build and assess mathematical models. Data for countries are from www.worldometers.info/coronavirus and for French administrative regions from www.statista.com/statistics/1101388/coronavirus-france-confirmed-cases. Data collection was constrained by the date of website accession—third week of March—and the availability of adequate data for specific countries at that date. This implies the inclusion of the first record with at least 100 cases after February 15, which excludes some countries for which the spread of the virus was more advanced at that period.

**Figure 1:**
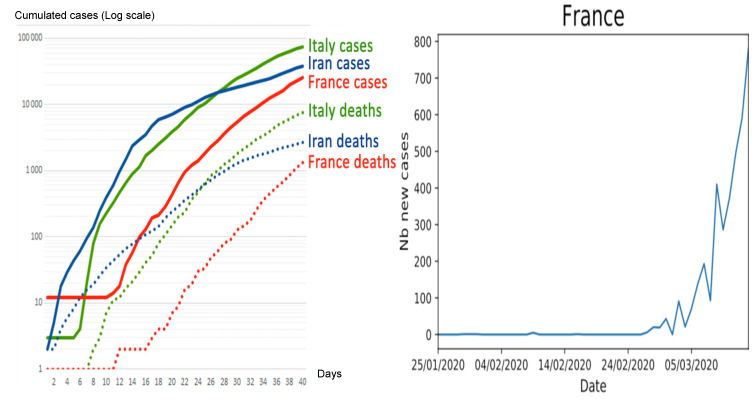
Left: Start of covid-19 epidemic in countries with various climates. Right: Daily number of new cases from 25 January until 14 March 2020 in France.

Figure 1 gives an example of data extracted from these databases. Epidemiological kinetics vary among countries, and could be due to differences in weather variables like temperature.

### 2.2. Modelling the Epidemic Spread with Temperature-Dependent Parameters

The classical epidemic modelling uses the continuous differential approach, which describes the infinitesimal change in the size of population of susceptible (*S*), infective (*I*) and recovering (*R*) individuals between times *t* and *t* + *dt.* This model explains the dynamics of epidemic spread, following the classical Bernoulli-d’Alembert-Ross equations [14]:

∂*S*/∂*t* = *rS* − *bSI* − *k*_1_*S* + *v_S_*∆*S*, ∂*I*/∂*t* = *bSI* − *k*_2_*I* − *k*’_2_*I* − *k*_1_*I* + *v*_*I*_∆*I*, ∂*R*/∂*t* = *k*′_2_*I* − *k*_1_*R* + *v*_*R*_∆*R*, (1)

where *r* is a renewal coefficient (depending on natality and immigration), *v*’s are diffusion coefficients (depending on population displacements), *b* is the contagion coefficient, which can depend on temperature, and the constants *k*_1_ and *k*_2_ are, respectively, the natural death rate and the specific epidemic death rate. An example of this continuous differential approach, applied to the covid-19 spread, has been developed by P. Magal [2], and more sophisticated approaches would take into account more general demographic (age-classes), sociologic (socio-economic categories), geographic (latitude and altitude) and climatic (temperature and humidity) variations explaining the heterogeneity of the data between the regions of the same countries and between the countries of the same geo-climato-demographic cluster. Some parameter dependencies are described in [14,15,16], like the dependency of *v* on altitude, *b* on temperature and humidity and *k’*s on age. The applications described in [14,16] have been performed, respectively, on classical Black Death data from St Anthony’s Order and Mali malaria data from O.K. Doumbo.

### 2.3. Statistical Time Series Modelling

The statistical time series modelling has been introduced by N. Wiener for prediction and forecasting [26]. Its parametric approach assumes that the underlying stationary stochastic process of the covid-19 new daily cases N(t) can be described using a small number of parameters using the autoregressive ARIMA model N(t) = Σ_i=1,s_ a(i) N(i) + W(t), where W is a random residue whose variance is to minimize. The autocorrelation analysis is done by calculating the correlation A(k) between the N(t)’s and N(t − k)’s (t belonging to a moving time window) by using the formula:

(2) $$A\left(k\right)=\frac{E\left[N\left(t\right)-E\left(N\left(t\right)\right)]E[N\left(t-k\right)-E\left(N\left(t-k\right)\right)\right]}{\sigma \left(N\left(t\right)\right)\sigma \left(N\left(t-k\right)\right)},$$

where E denotes the expectation and σ the standard deviation. The autocorrelation function A allows examining the serial dependence of the N(t)’s.

### 2.4. Statistical Analyses for French Administrative Regions and Selected Countries

Daily rates of new cases in French administrative regions and selected countries were correlated with mean temperatures in administrative capitals, using Pearson’s correlation coefficient r.

## 3. Results

### 3.1. Temperature Decreases Initial Negative Autocorrelation Slope of Epidemic Spread in Five Countries

**Figure 2:**
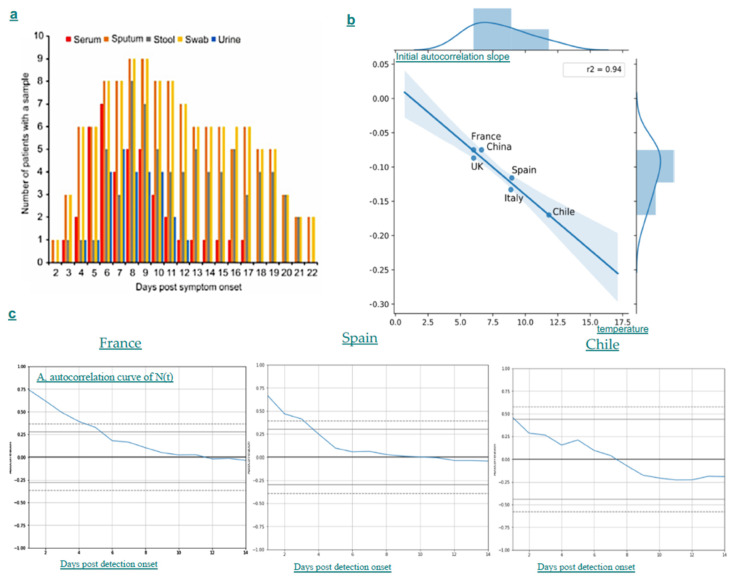
(**a**) Virulence of covid-19 in liquids and secretions (from [27]); (**b**) Linear regression of negative initial autocorrelation slope on mean weather temperature of six countries, France, UK, Spain, Italy, China and Chile (Pearson correlation coefficient R = 0.97, one-tailed *p* = 0.001). (**c**) Autocorrelation function A for three countries, France, Spain and Chile showing during February until 14 March 2020 a decrease in the positive correlation duration and the negative initial slope of the auto-correlation curve when the mean temperature of the country increases.

By using the classical ARIMA approach on the new cases’ time series in five countries with different weather (mild for France, Mediterranean for Italy, continental for Germany, oceanic for Chile and continental for China), we show that, for all countries, the regression minimizing the standard deviation (STD) of the residue W is of order 6 (Table 1), which is in agreement with the duration of the presence of virus in urine and blood after the mean incubation time of 6 days, which corresponds to the maximum of contagion for sputum, stool and swab (Figure 2a).

From the data of weather-atlas, we can calculate a mean temperature, obtained as the mean of the highest temperatures minus the mean of the lowest temperatures observed each day during February until 14 March 2020. The autocorrelation curves of the ARIMA regression have temperature-dependent shapes and the negative initial slope of their autocorrelation function decreases with the mean temperature (Figure 2b,c), reinforcing the plausibility of our hypothesis that the spread of covid-19 could diminish in warm weather, particularly at the start of the epidemic.

### 3.2. Temperature Decreases Regional Initial Rates of Epidemic Spread in France

**Figure 3:**
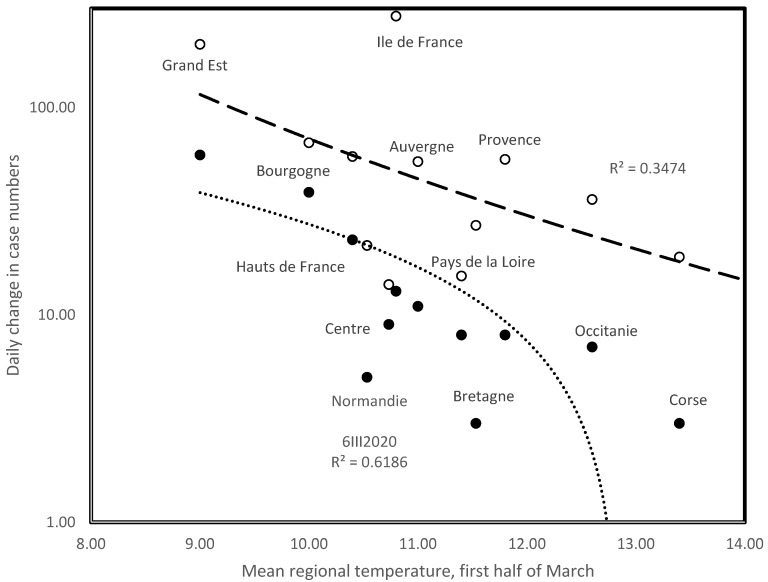
Daily increase in confirmed COVID-19 cases for administrative regions of France on 6 March 2020 (filled symbols, dotted line, log regression model) and on 15 March 2020 (circles, interrupted line, exponential regression model).

Table 2 presents the mean temperature in the first half of March in the 16 administrative regions of metropolitan France, numbers of confirmed covid-19 cases on 4 March, and ulterior daily rates as compared to data from the previous publication date. The last row shows Pearson correlation coefficients between regional rates of infection and mean temperatures across the period. Correlations show a slower increase in infection rates in warmer regions. The strength of this tendency decreases towards the end of this short period of rapid increase. This suggests that temperature most affects the early phases of epidemic dynamics. Figure 3 presents correlation analyses for two dates, 6 and 15 March 2020. Note that Figure 3 includes R^2^, which is the square of the Pearson correlation coefficient R. These adjustments are along logarithmic and exponential models for 6 and 15 March, respectively. We give the best (highest R^2^) models for these dates among linear (y = a.T + b), logarithmic (y = a.ln(T) + b), exponential (ln(y) = a.T + b) and power (ln(y) = a.ln(T) + b) regression models.

### 3.3. Temperature Decreases Country-Wise Initial Rates of Epidemic Spread

**Figure 4:**
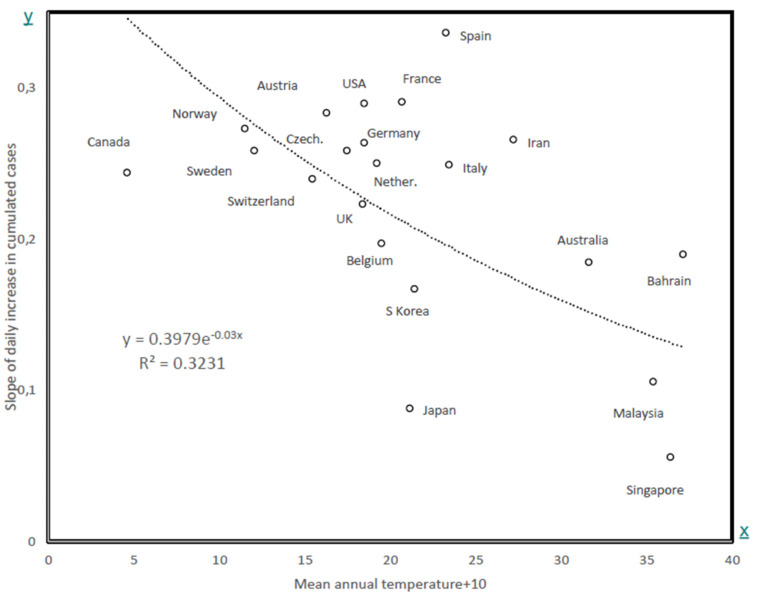
Slope of exponential model fitted to data in Table 3 as a function of mean annual temperature in that country. The Pearson correlation coefficient is R = −0.568, one-tailed *p* = 0.0036.

Table 3 presents the cumulative numbers of confirmed cases for countries with more than 100 cases in at least two days as of 14 March (last measure, besides for South Korea (10 March)). The constant and slope are for the exponential regression model to these data ln(N) = a × D + b, where D is the number of days since N crossed 100 confirmed cases in that country. Figure 4 shows the slope in the last column of Table 3 as a function of the mean annual temperature in that country. Slopes are lower and higher than expected by temperature for Canada and Spain, respectively. The causes for this might range from differences in containment policies and/or populational compliances, to other climatic factors (for example, humidity).

## 4. Discussion

A study (https://www.accuweather.com/en/health-wellness/coronavirus-expert-says-the-virus-will-burn-itself-out-in-about-6-months/679415) by J. Nicholls from the Sun Yat-sen University in Guangzhou, the capital of south China’s Guangdong province, has determined how the spread of the new coronavirus might be affected by changes in season and temperature.

We show in the present study that the negative initial slope of the autocorrelation curve related to the new daily cases N(t) of covid-19 spread and the duration of the positive autocorrelation period decreases when the weather temperature increases, which corresponds to a shorter duration of the period of contagiousness. The entropy of the distribution of daily R0’s during this period would decrease if the period becomes shorter, for the same overall R0. For example, if the daily R0’s tend to be the same (without peak), the entropy is maximum, equal to the logarithm of the number of contagious days. The decrease in the duration of the contagiousness period is considered favorable and corresponds to the "mitigation" of the contagion. The calculation of the entropy needs a precise estimation of the ARIMA coefficients a(i)’s in the development, N(t) = Σ_i=1,s_ a(i) N(i) + W(t), which is difficult during the transient start of the spread, due to the weak number of cases.

The two independent datasets, analysed at the level of French metropolitan administrative regions and of countries, show that temperature affects the increase in the number of cases at relatively local, as well as global, levels. The mechanisms by which temperature decreases the rates of detected cases are unknown. Higher temperatures might prevent the spread of droplets that transmit viruses, perhaps through faster evaporation. Other factors, like the decrease in the virus survival time in atmosphere probably also affect these rates, in particular at the level of the unexplained variation among countries shown in Figure 4. These results have to be considered with caution. They indicate that temperature affects early rates of spreading. It is unclear whether ulterior increases in seasonal temperatures will decrease rates [28,29,30,31]. Indeed, once a dynamic is set, temperature might not affect this dynamic anymore, or affect it only marginally.

Some papers on influenza show that if the warm weather period is followed by a cold season, the rebound of the epidemic could be severe, due to the loss of immune defense [8,15], and the present results have to serve as model for building a system of systematic surveillance along the future months of the covid-19 spread.

## 5. Conclusions

For more than fifty years, the relationship between weather and diseases has been studied [32]. Such studies are important for predicting viral disease spread, in particular if this leads to pandemics like in case of covid-19, in order to help decisions in public health policies at the world level.

## Figures and Tables

**Table 1:** ARIMA length of regression of new covid-19 cases on ambient temperature and standard deviation of the residue R(t) for five countries, France, Italy, Germany, Chile and China. (using https://www.statsmodels.org/stable/generated/statsmodels.tsa.arima_model.ARIMA.html)

Country	ARIMA (3,1,0) Residual STD	ARIMA (4,1,0) Residual STD	ARIMA (5,1,0) Residual STD	ARIMA (6,1,0) Residual STD	ARIMA (3,1,1) Residual STD
**France**	51.85	46.80	45.83	41.25	48.06
**Italy**	252.72	198.51		184.90	230.10
**Germany**	99.98	99.97	99.96	95.54	99.97
**Chile**	1.99	2.00		1.78	
**China**	361.52	344.30	343.50	342.61	349.15

**Table 2:** Dependence of the covid-19 new cases on temperature across administrative regions of Figure 2. Columns are: 1. Administrative region; 2. Mean temperature in the first half of March; 3. Numbers of confirmed cases on 4th of March; 4–10. Daily rate in change in number of cases vs previous date. Last row is the Pearson correlation coefficient R of above data in that column with mean temperature. For column 3, the latter correlation is not with a rate, as the date of first infection in that region varies among regions. All R < −0.47615 and R < −0.63385 have *p* < 0.05 and *p* < 0.01, respectively, according to one-tailed tests. Results indicate that negative effects of temperature on infection rates are strongest at the beginning of regional epidemics.

French Regions		2020	New Cases vs. Previous Day						
	Temp	4III	5III	6III	7III	10III	15III	23III	25III
Auvergne-Rhône-Alpes	11.00	49	15	11	27	49.0	54.8	150.9	181.5
Bourgogne-Franche-Comté	10.00	16	23	39	51	−2.0	67.6	110.8	111.0
Bretagne	11.53	23	6	3	8	14.3	27.0	34.0	56.5
Centre-Val de Loire	10.73	0	2	9	5	1.0	14.0	34.0	100.0
Corse	14.13	0	3	0	2	12.3	14.6	9.9	15.5
Grand Est	9.00	38	39	59	114	79.7	201.4	345.0	611.5
Hauts de France	10.40	65	9	23	76	25.3	58.0	91.3	242.0
Ile de France	10.80	55	21	13	15	121.3	275.6	545.6	724.5
Normandie	10.53	2	4	5	0	9.7	21.6	45.4	88.5
Nouvelle-Aquitaine	13.40	5	3	3	6	13.3	19.0	65.5	118.0
Occitanie	12.60	9	2	7	18	11.3	36.0	64.6	157.5
Pays de la Loire	11.40	7	1	8	2	4.3	15.4	23.1	37.5
Provence-Alpes-Côte d’Azur	11.80	13	5	8	12	24.0	56.2	139.9	208.5
Pearson Rx100		−48.95	−68.34	−74.73	−65.17	−34.3	−48.1	−43.5	−43.8

**Table 3:** Slope of exponential model fitted to data in Table 2 as a function of mean annual temperature in that country. The Pearson correlation coefficient is R = −0.568, one-tailed *p* = 0.0036.

Country/Day	1	2	3	4	5	6	7	8	9	10	11	12	13	14	15	16	17	18	19	20	Const	Slope
Australia	112	122	140	197																	88.138	0.1832
Austria	104	112	131	182	302	361	504														66.244	0.2825
Bahrain	109	110	189	195	210																88.724	0.1884
Belgium	109	169	200	239	267	314	314	599													102.14	0.1963
Canada	138	176																			108.2	0.2432
Czech Rep	116	150																			89.707	0.257
France	100	100	191	212	282	420	613	706	1116	1402	1774	2269	2860	3640							71.019	0.2898
Germany	129	157	196	262	534	639	795	1112	1139	1296	1567	2369	3062								106.46	0.2624
Iran	141	245	388	593	978	1501	2336	2922	3513	4747	5823	6566	7161	8042	9000	10,075	11,364				223.37	0.2641
Italy	124	229	322	400	650	888	1128	1689	2036	2502	3089	3858	4636	5883	7375	9172	10,149	12,462	15,113	17,660	169.95	0.2475
Japan	105	132	144	157	164	186	210	230	239	254	268	284	317	349	408	455	488	514	568	620	107.47	0.0872
Malaysia	117	129	129	129	197																100.72	0.1042
Netherlands	128	188	265	321	382	503	614	804													112.28	0.2485
Norway	113	147	169	192	277	489	489	750													79.017	0.2716
S Korea	104	204	346	602	763	977	1261	1766	2337	3150	3736	4212	4812	5328	5766	6284	6767	7134	7382	7513	323.41	0.1664
Singapore	102	106	108	110	110	117	130	138	150	160	166	178	187	200							90.377	0.0551
Spain	114	151	198	257	374	430	589	1024	1639	2140	2965	4231									71.126	0.335
Sweden	137	161	203	248	326	461	620	775													96.68	0.2572
Switzerland	209	264	332	332	491	645	858	1125													155.58	0.2388
UK	118	167	210	277	323	373	460	594	802												103.55	0.2223
USA	108	129	148	213	213	213	472	696	987	1264	1678										64.111	0.2882
